# A Nomogram Integrating Inflammatory Biomarkers and Echocardiographic Parameters for Predicting Left Ventricular Outflow Tract Obstruction Risk in Hypertrophic Cardiomyopathy

**DOI:** 10.3390/jcdd13090419

**Published:** 2026-08-27

**Authors:** Jinlei Li, Fen Ai, Yu Li, Bingxin Cheng, Zhen Chen

**Affiliations:** 1 Department of Emergency Medicine, The Central Hospital of Wuhan, Tongji Medical College, Huazhong University of Science and Technology, Wuhan 430014, China; 2 School of Medicine, Jianghan University, Wuhan 430056, China

**Keywords:** hypertrophic cardiomyopathy, left ventricular outflow tract obstruction, predictive model, neutrophil, nomogram

## Abstract

Objective: Although echocardiography is the gold standard for diagnosing left ventricularoutflow tract obstruction (LVOTO) in hypertrophic cardiomyopathy (HCM), relying solely on imaging is insufficient for precise risk stratification, particularly in borderline or atypical patients, and fails to capture systemic pathophysiological alterations. In recent years, novel inflammatory biomarkers derived from routine blood tests have shown sensitivity in capturing micro-inflammatory states; however, their specific roles in the obstructive phenotype of HCM remain unclear. This study aims to screen hematological and cardiac parameters associated with HCM obstruction and to construct an individualized predictive model. Therefore, this study aims to screen hematological and cardiac parameters associated with HCM obstruction and to develop and validate a nomogram for individualized prediction of current LVOTO risk in HCM patients. Methods: A total of 230 HCM patients hospitalized at The Central Hospital of Wuhan from January 2019 to December 2025 were retrospectively enrolled. Based on the left ventricular outflow tract pressure gradient (LVOTPG), they were divided into a non-obstruction group (*n* = 177) and an obstruction group (*n* = 53). Least absolute shrinkage and selection operator (LASSO) regression was used to screen feature variables, and multivariate logistic regression analysis was employed to identify independent predictors and construct a nomogram prediction model. The discrimination, calibration, and clinical utility of the model were evaluated using receiver operating characteristic (ROC) curves, calibration curves, and decision curve analysis (DCA). Results: Multivariate logistic regression analysis revealed that a history of alcohol consumption (OR = 3.68, 95% CI: 1.49–9.09), peak LVOTPG (OR = 3.96, 95% CI: 1.42–11.05), maximum ventricular wall thickness (OR = 1.02, 95% CI: 1.00–1.04), peak mitral valve E-wave velocity (OR = 1.02, 95% CI: 1.00–1.04), and neutrophil count (OR = 2.42, 95% CI: 1.13–5.21) were independent predictors of LVOTO in HCM patients. The nomogram model constructed based on these factors achieved area under the curve (AUC) values of 0.810 and 0.850 in the training and validation sets, respectively. The calibration curve demonstrated good agreement between predicted and actual probabilities, and DCA indicated that the model provided clinical net benefit within a threshold probability range of 10% to 30%. Conclusions: A nomogram integrating alcohol consumption history, peak LVOTPG, maximum ventricular wall thickness, peak mitral E-wave velocity, and neutrophil count accurately predicts LVOTO risk in HCM patients (AUC: 0.810–0.850), providing a low-cost tool for early risk stratification. External prospective validation is warranted.

## 1. Introduction

Hypertrophic cardiomyopathy (HCM) is a genetic myocardial disease characterized by asymmetric myocardial hypertrophy, with an incidence rate of approximately 1 in 500, and is one of the leading causes of sudden cardiac death (SCD) in adolescents and athletes [1]. According to the 2023 European Society of Cardiology (ESC) guidelines, the diagnostic criteria for HCM are primarily based on a left ventricular (LV)myocardial wall thickness of ≥15 mm, with myocardial hypertrophy not solely explained by loading conditions [2]. The clinical manifestations of HCM patients are diverse, and approximately 25% of HCM patients present with LV outflow tract obstruction (LVOTO). This phenotype significantly increases the risk of sudden cardiac death (SCD) and is a key focus of clinical management [3].

The diagnosis of HCM primarily relies on echocardiography, and treatment focuses on symptom control and complication prevention [4]. In recent years, chronic low-grade inflammation has been recognized not merely as an accompanying phenomenon, but as a core mechanism driving myocardial remodeling and influencing prognosis [5,6]. Myocardial histological analysis has confirmed that the degree of inflammatory cell infiltration is significantly positively correlated with the degree of fibrosis [7].

In biomarker research, most studies have focused on single inflammatory markers such as hs-CRP [8], NT-proBNP [9], or IL-6 [10], with limited specificity and predictive value. Recently, novel composite markers such as NLR [11,12], SII [13], and MHR [14,15,16] have been proposed to more comprehensively reflect immune imbalance. However, few studies have targeted predictive markers specifically for the progression from non-obstructive to obstructive HCM. Notably, the Fuwai Hospital team recently defined an inflammatory subtype characterized by “high LVOTPG accompanied by significant inflammatory cell infiltration” [17], highlighting the clinical need for precise identification of high-risk subtypes.

Additionally, the progression of HCM from the non-obstructive to the obstructive form is a chronic process, with patients remaining asymptomatic and the condition often incidentally discovered during routine echocardiographic screening. Although echocardiography is the current routine method for diagnosing LVOTO in hypertrophic cardiomyopathy, it has limitations. First, frequent ultrasound follow-ups for asymptomatic patients are costly and not significantly cost-effective [18]. Moreover, echocardiography is operator-dependent, carrying the risk of diagnostic errors, particularly in complex HCM cases where differentiating true obstruction from physiological murmurs is challenging [19]. Some gene mutation carriers may present with borderline or normal wall thickness, leading to insufficient warning of SCD risk [20]. Second, LVOTO is a highly dynamic hemodynamic phenomenon; measuring it only at rest may underestimate obstruction in up to 60–75% of patients who only develop significant obstruction during exercise [21]. Furthermore, asymptomatic patients often struggle to understand the need for repeated “normal” tests, and this cognitive gap can undermine adherence to long-term follow-up [22].

We hypothesized that: (1) novel inflammatory biomarkers derived from routine blood tests are independently associated with the presence of LVOTO in HCM patients, beyond traditional echocardiographic parameters; and (2) a nomogram integrating these inflammatory biomarkers with echocardiographic and clinical variables can provide superior predictive performance for identifying HCM patients at high risk of obstruction, particularly in borderline or diagnostically challenging cases.

This study aims to: (1) Systematically screen and evaluate the association of various novel inflammatory markers with the obstructive phenotype of HCM, and identify the core inflammatory markers with the highest predictive value. (2) Screen clinically accessible hematological and basic clinical features to construct and validate a nomogram prediction model for HCM obstruction risk. This model not only aims to provide a low-cost, convenient early risk stratification tool for borderline populations with diagnostic difficulties, but also aspires to serve as a biological window reflecting disease activity, providing new insights and evidence for future exploration of individualized treatment strategies targeting inflammatory pathways.

## 2. Materials and Methods

### 2.1. Study Population

This study retrospectively enrolled 230 patients diagnosed with HCM who were hospitalized at The Central Hospital of Wuhan from January 2019 to December 2025. The diagnosis of HCM was based on the 2023 ESC Guidelines [2], defined as unexplained LV myocardial wall thickness ≥15 mm in one or more segments, not solely explained by abnormal loading conditions.

Exclusion criteria included: (1) hypertension or valvular heart disease sufficient to explain the degree of myocardial hypertrophy; (2) concurrent malignant tumors, acute infections, autoimmune diseases, or hematologic disorders; (3) missing key clinical data.

After applying the above exclusion criteria, all remaining patients were confirmed HCM cases hospitalized in the Department of Cardiology at our center. Reasons for admission encompassed initial diagnostic evaluation, symptom exacerbation, coronary angiography assessment to rule out concomitant coronary artery disease, and routine follow-up.

According to the resting LVOTPG, patients were divided into a non-obstruction group (LVOTPG < 30 mmHg, *n* = 177) and an obstruction group (LVOTPG ≥ 30 mmHg, *n* = 53). This study was approved by the Ethics Committee of The Central Hospital of Wuhan, and informed consent was waived due to its retrospective nature.

### 2.2. Sample Size Estimation

The sample size for this study was estimated based on previous literature and preliminary data from our hospital, with LVOTO in HCM patients as the primary outcome. According to our preliminary data, the estimated proportion of heart failure cases among all HCM patients was 25%, with an allowable margin of error of 5%. Using a two-sided significance level of α = 0.05 and a statistical power of 1-β = 0.90, the minimum calculated sample size was 181 cases. To ensure adequate statistical power for the internal validation of the model, an additional 25% of cases were enrolled, resulting in a cumulative total of 230 patients. The study was conducted in accordance with the Declaration of Helsinki, and approved by the Ethics Committee of Wuhan Central Hospital (protocol code WHZXKYL2025-255, date of approval: 23 December 2025).

### 2.3. Data Collection

To clarify, this study utilized data from each patient’s initial hospitalization period. For patients with multiple admissions, only data from their first hospitalization for HCM-related diagnosis or treatment at our center were extracted. All clinical data were extracted from the electronic medical record system of the Biobank at The Central Hospital of Wuhan. The extracted data included basic demographic characteristics (age, sex, body mass index (BMI), etc.), comorbidities (hypertension, diabetes, etc.), biochemical parameters (blood lipids, complete blood count (CBC), etc.), and imaging examination results upon admission. Novel inflammatory biomarkers were calculated based on the biochemical parameters. According to the echocardiographic findings, patients were divided into a non-obstruction group (LVOTPG < 30 mmHg) and an obstruction group (LVOTPG ≥ 30 mmHg) [3].

**Figure 1:**
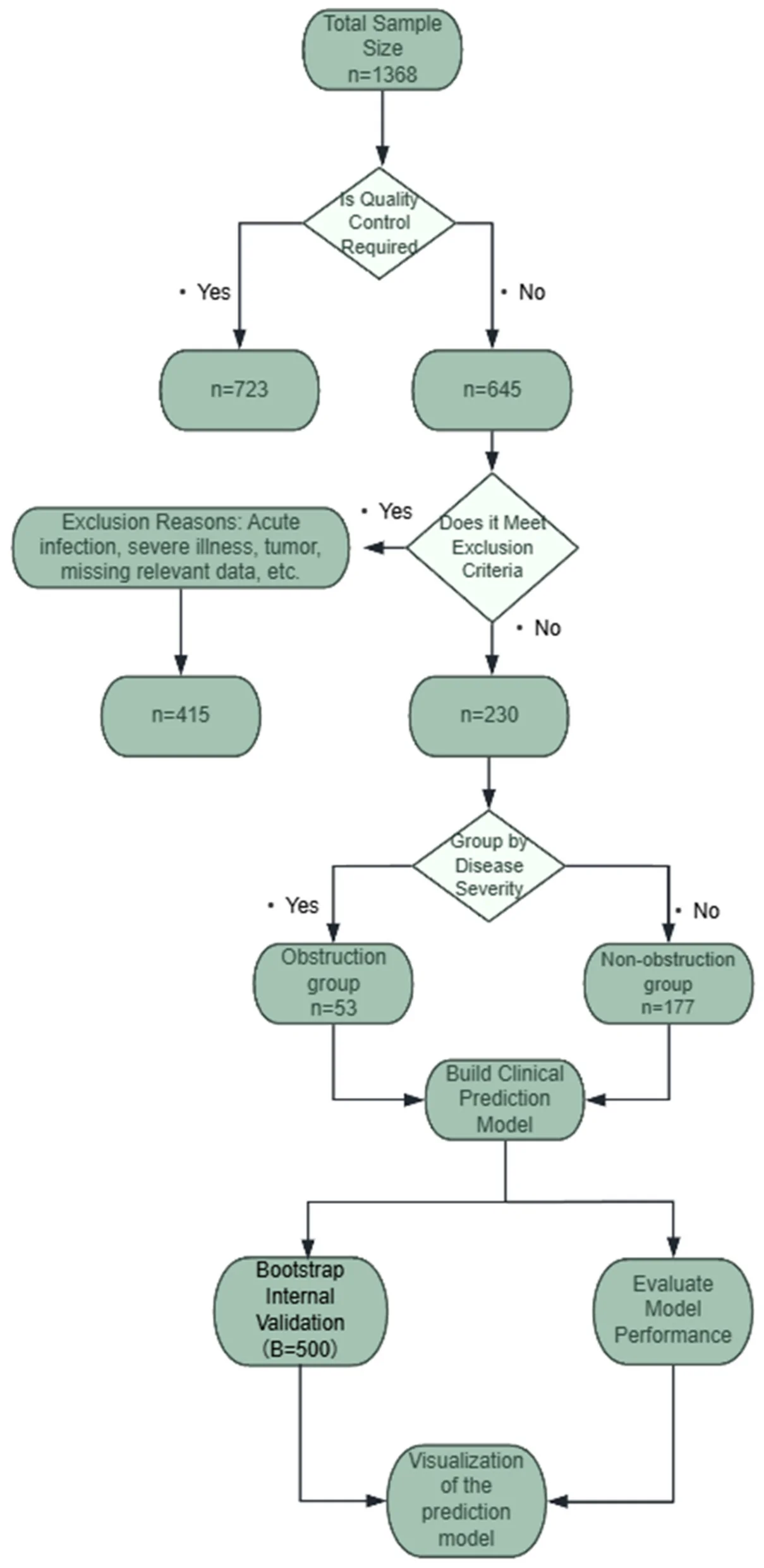
Flowchart of the development and validation of the hypertrophic cardiomyopathy prediction model.

A total of 1368 patients hospitalized with HCM at The Central Hospital of Wuhan between January 2019 and December 2025 were initially enrolled in this study. After screening, 723 cases of readmission were excluded, leaving 645 cases. Based on the exclusion criteria, a final cohort of 230 patients was included, comprising 53 patients in the obstruction group and 177 patients in the non-obstruction group (Figure 1).

In this study, we prioritized several novel composite inflammatory markers that have garnered increasing attention in the cardiovascular field in recent years. Compared with traditional single inflammatory markers (e.g., hs-CRP), these markers integrate information on innate immunity (neutrophils, monocytes) and adaptive immunity (lymphocytes), combined with dimensions of lipid metabolism (HDL-C) or thrombotic tendency (platelets). Theoretically, they may more comprehensively characterize the systemic inflammatory microenvironment associated with HCM. Furthermore, all markers can be calculated from routine laboratory test results, demonstrating good potential for clinical translation.

The novel inflammatory biomarkers included in this study were defined as follows:

NHR: Neutrophil-to-High-Density Lipoprotein Cholesterol Ratio

MHR: Monocyte-to-High-Density Lipoprotein Cholesterol Ratio

LHR: Lymphocyte-to-High-Density Lipoprotein Cholesterol Ratio

MLR: Monocyte-to-Lymphocyte Ratio

LMR: Lymphocyte-to-Monocyte Ratio

PHR: Platelet-to-High-Density Lipoprotein Cholesterol Ratio

SII: Systemic Immune-Inflammation Index = (Neutrophil count × Platelet count)/Lymphocyte count

SIRI: Systemic Inflammation Response Index = (Neutrophil count × Monocyte count)/Lymphocyte count

AISI: Aggregate Index of Systemic Inflammation = (Neutrophil count × Monocyte count × Platelet count)/Lymphocyte count

### 2.4. Statistical Analysis

All statistical analyses were performed using R software (version 4.4.2). A two-sided *p*-value < 0.05 was considered statistically significant. Normally distributed continuous variables were expressed as mean ± standard deviation (SD) and compared between groups using the independent samples *t*-test. Non-normally distributed data were presented as median (interquartile range, IQR) and compared using the Mann–Whitney *U* test. Categorical variables were expressed as frequencies and percentages [*n* (%)] and compared using the chi-square (χ^2^) test or Fisher’s exact test, as appropriate.

### 2.5. Predictor Selection and Model Construction

To prevent overfitting of high-dimensional data, Least Absolute Shrinkage and Selection Operator (LASSO) regression was performed using the glmnet package (4.1-10) in R to screen all candidate variables. The optimal penalty coefficient (λ) was determined through 10-fold cross-validation. To enhance the generalizability of the model, the variable set corresponding to “lambda.1se” was selected.

The variables selected by LASSO were subsequently incorporated into univariate logistic regression. Variables with a *p*-value <0.1 in univariate analysis were retained for multivariate logistic regression. The final independent diagnostic predictors were identified using forward stepwise regression based on the Akaike Information Criterion (AIC). Variables with a *p*-value <0.05 were retained in the final nomogram. Variables with a *p*-value between 0.05 and 0.1 were reported in the tables but were not included in the nomogram, while variables with a *p*-value ≥0.1 were excluded.

Subsequently, based on the results of the multivariate logistic regression, a nomogram was developed using the rms package (8.1-1). Furthermore, an interactive dynamic nomogram was created using the DynNom package (5.1) to improve the convenience and clinical utility of the model.

### 2.6. Model Performance Evaluation and Validation

Internal validation was performed using 500 bootstrap resamples. Specifically, 500 Bootstrap samples, each with a sample size of 230, were randomly drawn with replacement from the original dataset (*n* = 230). The model was refitted on each Bootstrap sample and then evaluated on the original dataset. The discriminatory performance of the training and validation sets was compared using the DeLong test. The model’s discrimination, calibration, clinical feasibility, and rationality were evaluated separately.

Discrimination was assessed by plotting receiver operating characteristic (ROC) curves and calculating the area under the curve (AUC). An AUC value between 0.70 and 0.80 indicates moderate discrimination, while an AUC value >0.80 indicates high discriminatory ability. The optimal cutoff value was determined using the Youden index, and sensitivity and specificity were subsequently calculated.

Calibration curves and the Hosmer-Lemeshow goodness-of-fit test were used to evaluate the agreement between predicted and actual probabilities. The Brier score and *p*-value (*p* > 0.05 indicating good calibration) were calculated to quantitatively assess calibration performance. Finally, decision curve analysis (DCA) was performed to evaluate the net clinical benefit of the model across different threshold probabilities.

## 3. Results

### 3.1. Demographic and Baseline Clinical Characteristics

The demographic and baseline clinical characteristics of the two groups are summarized in Table 1. No statistically significant differences were observed between the two groups regarding sex distribution, age, or the prevalence of comorbidities such as hypertension, diabetes mellitus, and coronary heart disease. Furthermore, the usage rates of commonly prescribed cardiovascular medications, including ARBs, beta-blockers, CCBs, and diuretics, were well-balanced between the groups.

However, patients in the obstruction group exhibited a significantly higher BMI and a greater prevalence of smoking and alcohol consumption compared to those in the non-obstruction group. Additionally, although the difference in age did not reach statistical significance, patients in the obstruction group tended to be relatively younger.

### 3.2. Analysis of Blood and Lipid Parameters

This study further compared the CBC and lipid metabolism parameters between the two groups (Table 2). Most traditional CBC parameters and lipid profiles showed no significant differences between the two groups, except for platelet count (*p* = 0.031) and HDL-C (*p* = 0.01), which were significantly higher and lower, respectively, in the obstruction group.

These findings underscore the necessity of further exploring novel inflammatory biomarkers that integrate information on immune activation and metabolic dysregulation, aiming to more precisely elucidate the underlying risk factors for LVOTO.

### 3.3. Analysis of Echocardiographic Structural and Functional Parameters

As the gold standard for evaluating the obstructive phenotype of hypertrophic cardiomyopathy (HCM), echocardiographic parameters demonstrated significant intergroup specificity in this study (Table S1). In our cohort, the hypertrophy in the obstructive HCM group was predominantly distributed in the LV posterior wall and basal segments. Consequently, there was no significant intergroup difference in interventricular septal thickness, whereas the maximum wall thickness and LV posterior wall thickness were significantly greater in the obstruction group.

### 3.4. Analysis of Novel Inflammatory Biomarkers

This study further focused on novel inflammatory biomarkers capable of integrating information on immune activation and metabolic dysregulation (Table 3). The results showed that NHR was the only indicator that exhibited a statistically significant difference between the obstruction and non-obstruction groups, followed by MLR, which was marginally significant. Although the values of the remaining indicators (LMR, PHR, SII, SIRI, and AISI) were numerically higher in the obstruction group, the differences did not reach statistical significance.

### 3.5. Screening of Predictors

**Figure 2:**
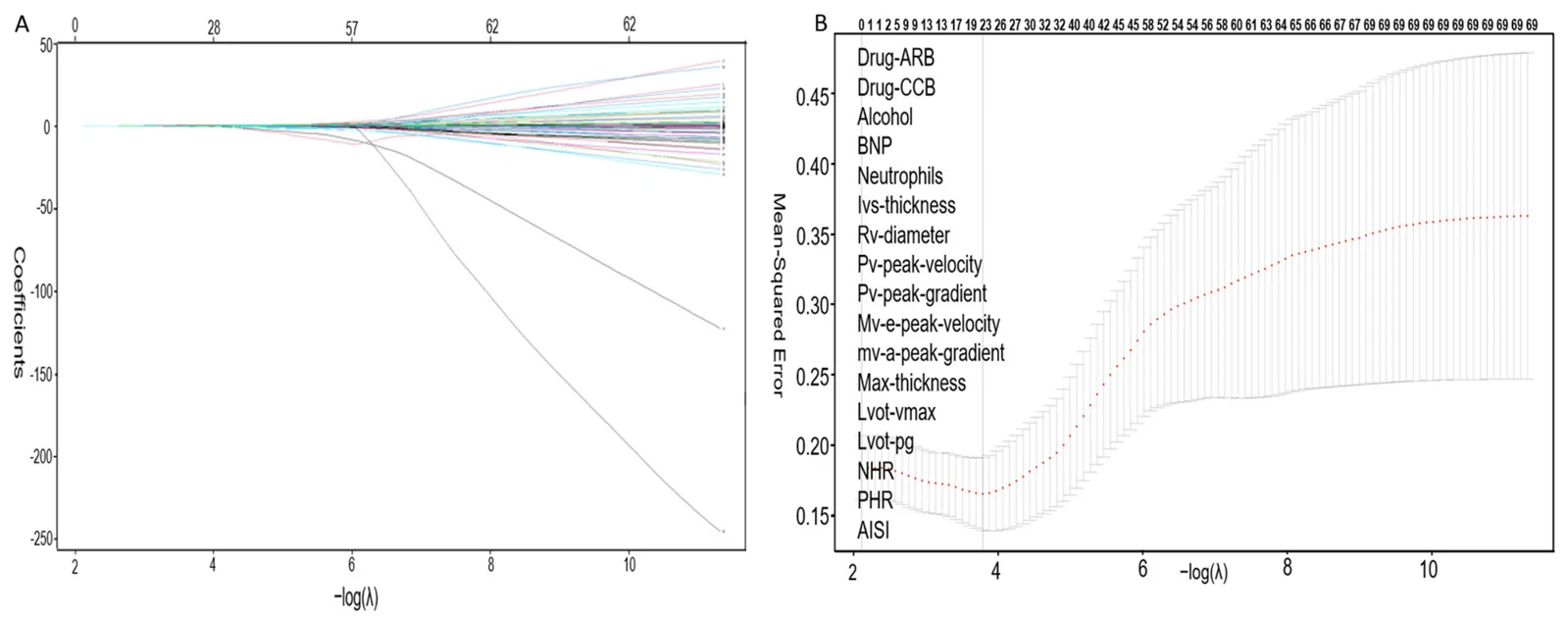
Feature selection process using the LASSO regression model in the training cohort. (**A**) The LASSO coefficient profiles obtained from the regression analysis. (**B**) Ten-fold cross-validation was performed to select the optimal λ value of 0.018. The x-axis represents the optimal tuning parameter (Log λ), and the y-axis represents the binomial deviance. The dashed lines indicate the positions of lambda.min and lambda.1se.

To select the most predictive factors from numerous candidate variables and prevent model overfitting, LASSO regression was employed for preliminary feature selection. As the logarithm of the regularization parameter (λ) increased, the regression coefficients of the variables gradually approached zero (Figure 2A). The optimal λ value (λ = 0.018) was determined via 10-fold cross-validation, and the “lambda.1se” criterion was selected to obtain a more parsimonious model.

A total of 16 variables with non-zero coefficients were identified as the core feature set for subsequent analyses. These included ARBs, CCBs, a history of alcohol consumption, BNP, neutrophil count, right ventricular dimension, peak pulmonary vein flow velocity, peak pulmonary vein pressure gradient, peak mitral E-wave velocity, peak mitral A-wave velocity, maximum wall thickness, peak LVOT velocity, peak LVOTPG, PHR, NHR, and AISI.

Subsequently, these 16 potential diagnostic indicators were incorporated into a multivariate logistic regression model for analysis (Table S2). Using a *p*-value < 0.1 as the inclusion criterion and *p* < 0.05 as the retention criterion, a history of alcohol consumption, peak LVOTPG, maximum wall thickness, peak mitral E-wave velocity, and neutrophil count were ultimately identified as independent predictors of LVOTO in patients with HCM (Table 4).

Additionally, PHR (*p* = 0.060) and peak pulmonary valve pressure gradient (*p* = 0.088) also showed suggestive significance in the multivariate regression; however, they were not included in the final nomogram because they did not meet the predefined threshold for statistical significance. Ultimately, these five variables were established as the fundamental indicators for developing the predictive model.

### 3.6. Construction of the Nomogram

**Figure 3:**
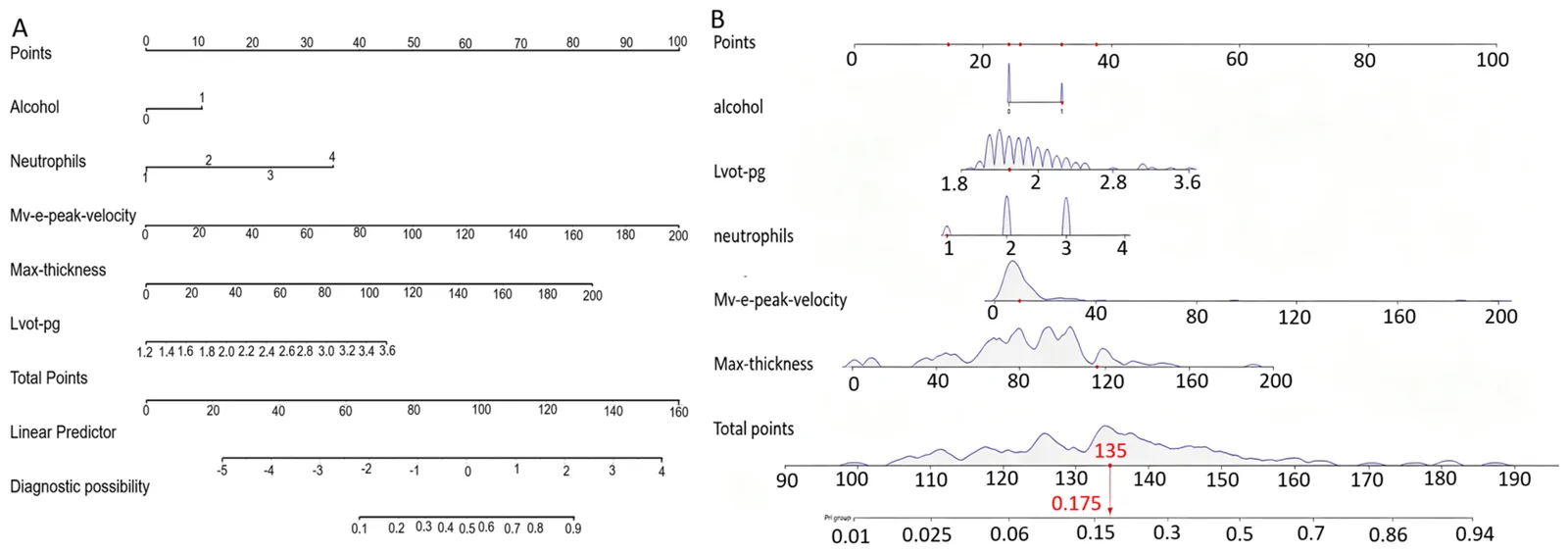
Nomogram and dynamic nomogram for predicting LVOTO risk in HCM patients. (**A**) Static nomogram integrating five independent predictors to estimate individual LVOTO probability. (**B**) Interactive dynamic nomogram displaying predictor density distributions and real-time predicted probability based on total points.

Based on the five predictors identified through multivariate logistic regression analysis, a nomogram model was constructed to individually predict the risk of LVOTO in patients with HCM (Figure 3A). This model translates the regression coefficients from the multivariate logistic regression into a user-friendly scoring system. Each predictor is assigned a score based on its relative contribution, and the total score corresponds directly to the individual probability of developing LVOTO.

To enhance the clinical utility of the model, an interactive dynamic nomogram was created (Figure 3B). This tool allows for the real-time input of patient-specific parameters, facilitating the immediate calculation and visualization of risk probabilities, thereby improving the convenience and flexibility of clinical decision support.

### 3.7. Model Performance Evaluation

**Figure 4:**
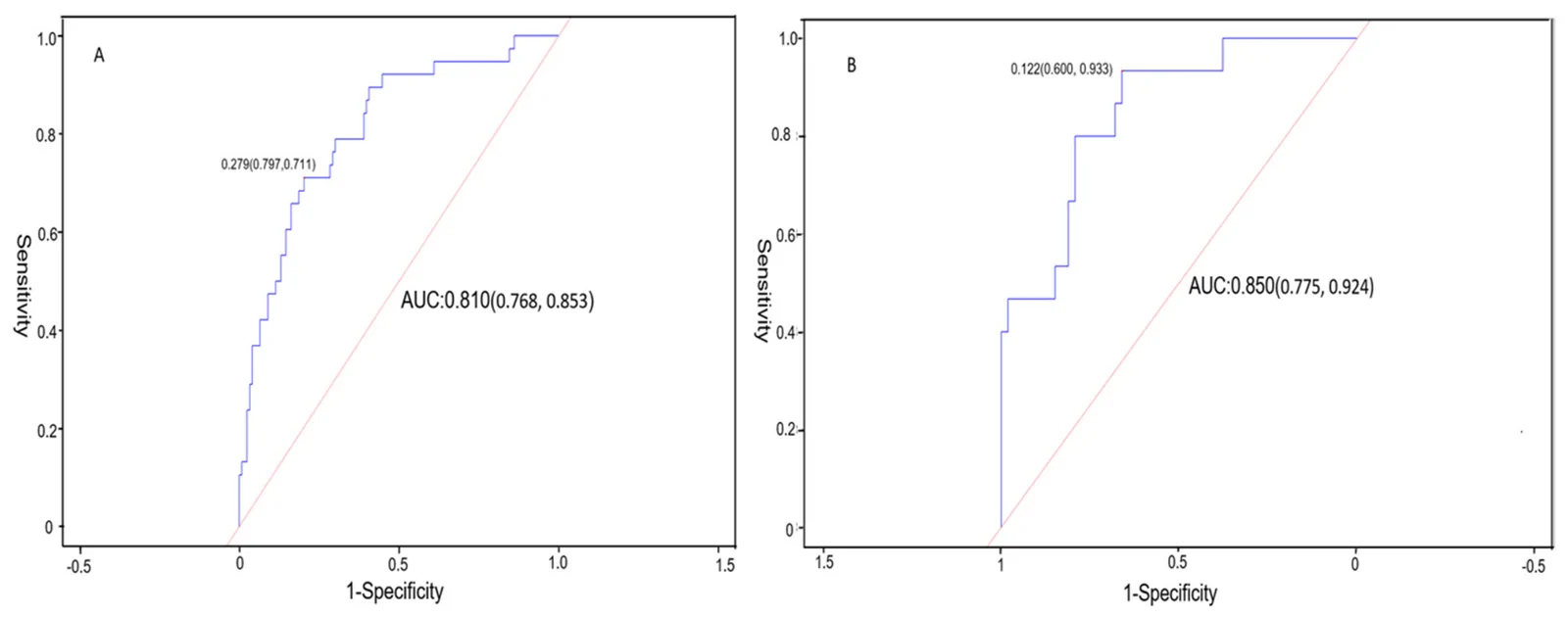

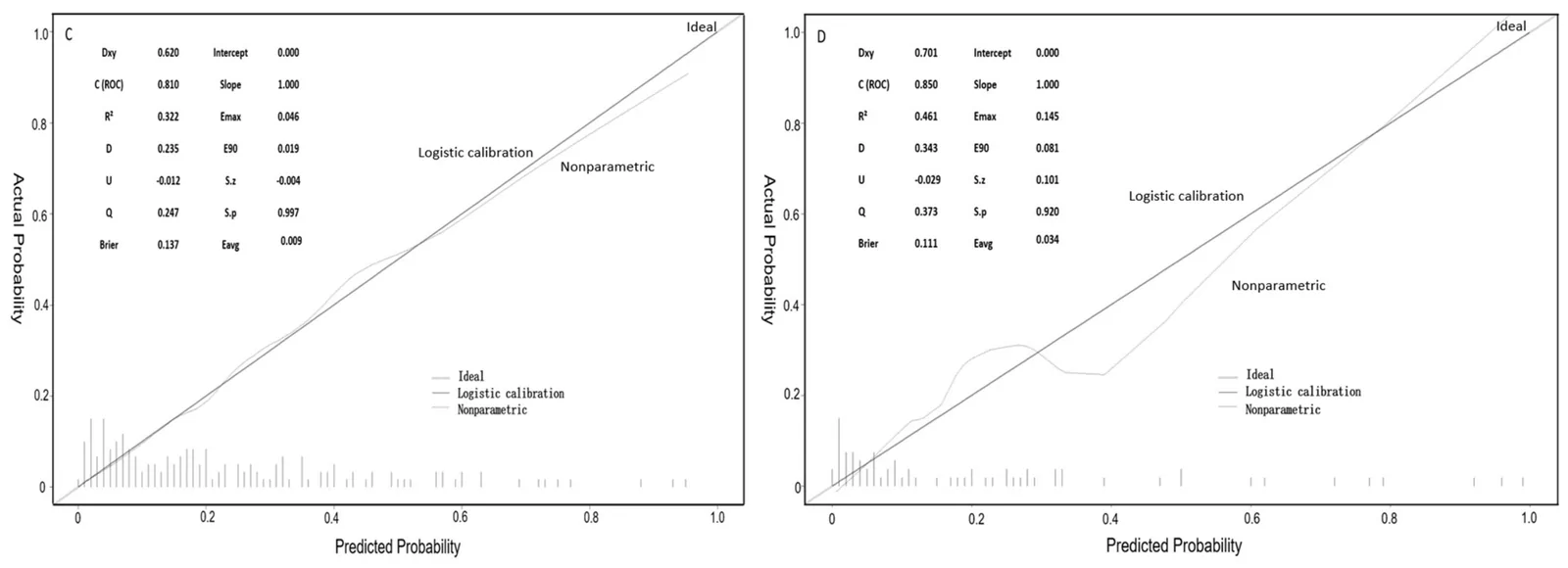
Discriminative and calibration performance of the model. (**A**,**B**) Receiver operating characteristic (ROC) curves for the training and validation cohorts, respectively. (**C**,**D**) Calibration curves for the training and validation cohorts, respectively. The x-axis represents the predicted probability of LVOTO in HCM, and the y-axis represents the actual observed incidence of LVOTO in HCM. The gray diagonal line indicates perfect agreement between the predicted and actual probabilities.

**Figure 5:**
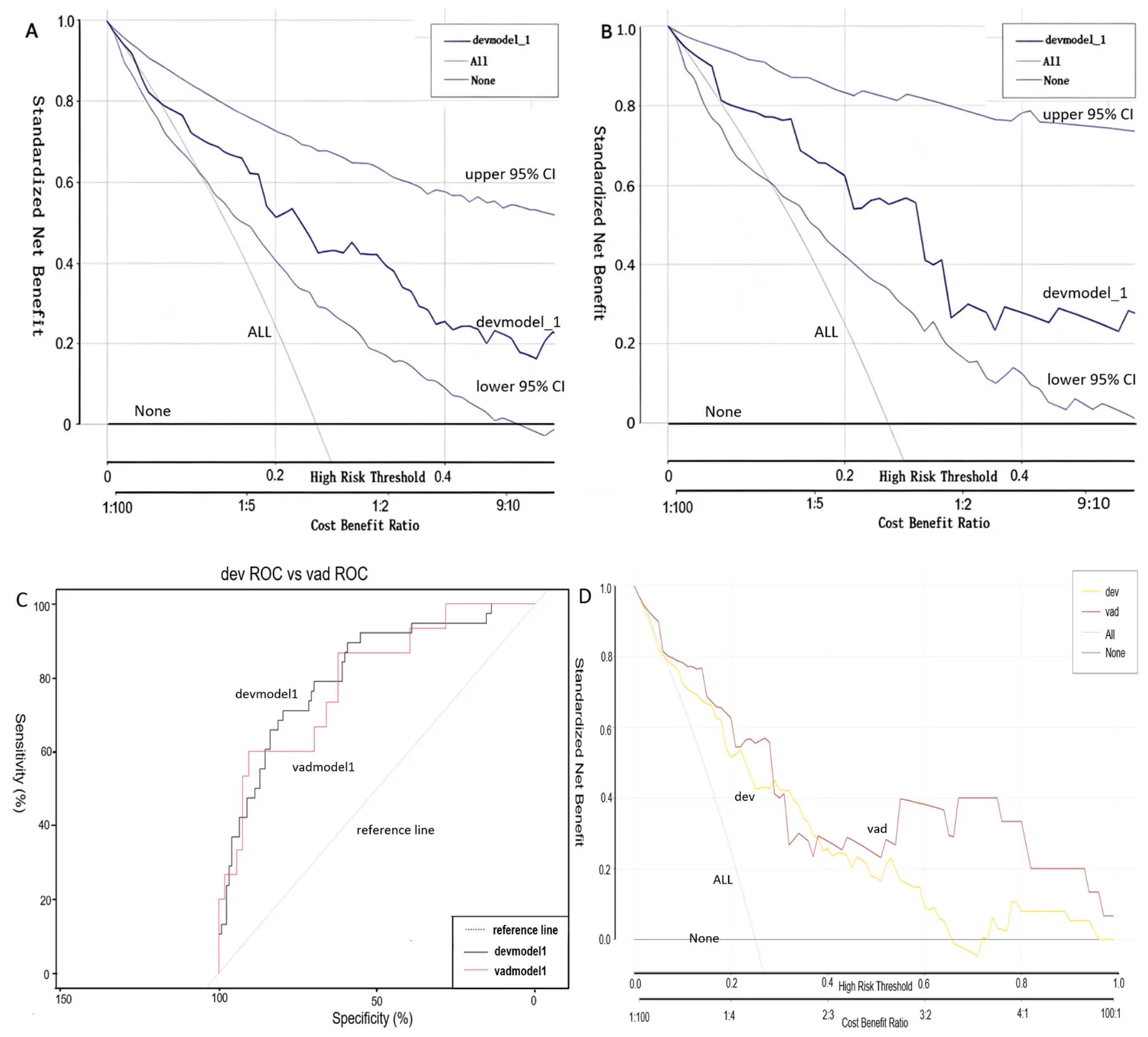
Decision curve analysis (DCA) for the training and validation cohorts. The x-axis represents the threshold probability, and the y-axis represents the net benefit. In (**A**), the decision curve of the training cohort demonstrates the model’s clinical net benefit. In (**B**), the decision curves of the validation cohort demonstrate strong consistency with the training cohort, indicating the generalizability of the model’s net clinical benefit. In (**C**), the ROC curves of the training and validation cohorts are highly overlapping, indicating consistent discriminatory performance of the model. In (**D**), the decision curves of the training and validation cohorts demonstrate strong consistency, indicating the generalizability of the model’s net clinical benefit.

The model demonstrated excellent discriminatory performance in both the training and validation cohorts, with areas under the ROC curves and calculating the AUC of 0.810 and 0.850, respectively (Figure 4). At the optimal diagnostic threshold, the model exhibited favorable sensitivity and specificity. Calibration curves showed a high degree of agreement between the predicted and actual observed probabilities, fitting the ideal diagonal line well (Figure 5). The Hosmer-Lemeshow goodness-of-fit test yielded no statistically significant results (*p* > 0.05), and the training set exhibited a low Brier score, further confirming the excellent calibration of the model.

Decision curve analysis (DCA) indicated that when using this model for clinical decision-making, both the training and validation cohorts demonstrated significant net clinical benefits within a threshold probability range of 10% to 30% (Figure S1). Rational analysis further confirmed that this comprehensive model outperformed individual indicators in both discriminatory ability (ROC curves) and net clinical benefit (decision curves), highlighting the additional diagnostic value of the multivariate integrated model.

Furthermore, the stability of the model performance metrics was validated through 500 bootstrap resamples. The DeLong test revealed no statistically significant difference in AUC between the training and validation cohorts. Moreover, both cohorts exhibited highly consistent standardized net benefit curves, demonstrating the model’s robustness and its generalizability in internal validation.

## 4. Discussion

In this retrospective case–control study, we systematically investigated the associations of hematological parameters, novel inflammatory markers, and echocardiographic parameters with the risk of LVOTO in patients with HCM. We found significant differences in multiple indicators between the obstruction and non-obstruction groups. Patients in the obstruction group not only had a higher BMI and a greater proportion of smokers and alcohol consumers, but their hematological profiles also exhibited more pronounced features of inflammation and metabolic dysregulation, characterized by higher platelet counts, lower HDL-C levels, and a significantly elevated NHR. Furthermore, echocardiographic results confirmed that the obstruction group had significantly greater maximum wall thickness, LV posterior wall thickness, and various hemodynamic parameters, reflecting more severe hemodynamic impairment.

Based on these findings, LASSO regression and multivariate logistic regression analyses identified five independent predictors: a history of alcohol consumption, peak LVOTPG, maximum wall thickness, peak mitral E-wave velocity, and neutrophil count. These predictors comprehensively reveal the complex mechanisms underlying LVOTO in HCM patients from multiple dimensions, encompassing lifestyle, inflammatory status, and cardiac structure and function. As a risk factor, a history of alcohol consumption may potentially exacerbate the outflow tract pressure gradient through its vasodilatory effects, although the precise mechanisms remain to be elucidated. Neutrophil count may reflect the involvement of systemic inflammation in myocardial remodeling and fibrosis, raising the possibility that inflammatory responses could contribute to the evolution of the obstructive phenotype, though further mechanistic studies are needed [23]. Regarding cardiac structure, maximum wall thickness serves as the anatomical basis for obstruction; its thickening not only directly causes outflow tract narrowing but also creates the conditions for SAM of the mitral valve. As a hemodynamic indicator, the baseline peak LVOTPG quantifies the potential risk of obstruction. Even if the gradient does not reach the diagnostic threshold, its elevation indicates that the patient is in a “borderline obstruction” state [24]. Finally, as a key parameter of diastolic function, an abnormal mitral E-wave velocity reflects increased ventricular stiffness and diastolic dysfunction, which are closely associated with the onset and progression of obstruction.

It must be acknowledged that our study focused on the association between inflammation and LVOTO, but did not examine its potential role in driving progression to a dilated/decompensated heart failure phenotype or arrhythmic events. This is a notable limitation, as increased myocardial inflammation and fibrosis may also contribute to these adverse outcomes.

Admittedly, the obstructive phenotype is often associated with more severe disease burden. However, our model is not simply equivalent to disease staging. Neutrophil count, as a predictor independent of maximum wall thickness and LVOTPG, points to an additional, active biological process—systemic inflammation. We hypothesize that this subset of patients may represent the aforementioned “inflammatory subtype,” in which active inflammatory processes within the myocardial microenvironment actively drive the transition from non-obstructive to obstructive phenotypes. Therefore, this model may help identify individuals who, even at an early stage of disease, are at risk of rapid progression due to a heightened inflammatory state.

We acknowledge that classic sarcomeric HCM is fundamentally a genetic disorder rather than an inflammatory disease. However, due to the retrospective design, genetic testing results were not available for all patients, and we could not definitively distinguish between sarcomeric HCM and phenocopies (e.g., Fabry disease, cardiac amyloidosis). Although we excluded patients with hypertension or valvular heart disease sufficient to explain the degree of hypertrophy, the possibility of including undiagnosed phenocopies cannot be entirely excluded. Emerging evidence suggests that chronic low-grade inflammation acts as a disease modifier in sarcomeric HCM, accelerating myocardial fibrosis and adverse remodeling [5,6]. Future prospective studies incorporating genetic screening are warranted to validate the applicability of this nomogram across different HCM etiologies.

The core clinical value of this model lies in its role as a convenient, low-cost screening tool to help clinicians rapidly identify HCM patients at high risk of obstruction in outpatient or inpatient settings. For example, in patients with ‘borderline’ obstruction on echocardiography, combining the model’s score may facilitate more decisive decisions regarding the need for stress echocardiography or initiation of targeted therapy, thereby enabling early intervention.

To further illustrate the clinical utility of the nomogram, we propose the following phenotypic scenarios. First, for a patient with borderline resting LVOTPG (15–29 mmHg), a high nomogram score would prompt consideration of stress echocardiography or early pharmacological intervention. Second, for an asymptomatic patient with normal resting gradients but a high nomogram score, the model may identify an “inflammatory subtype” at risk of rapid progression, justifying intensified surveillance and lifestyle modification. Third, a low nomogram score in a patient with mild hypertrophy and no inflammatory features may support a conservative approach, avoiding unnecessary testing. These examples demonstrate how the nomogram can guide individualized management beyond traditional echocardiographic assessment.

This study has certain limitations. First, as a single-center retrospective analysis with a relatively limited sample size (particularly in the obstruction group), it may be subject to selection bias. The lack of validation in an independent external cohort also limits the generalizability of the model to broader populations. Second, although multiple confounding factors were adjusted for, the interference of unmeasured variables, such as medication use and volume status, cannot be completely excluded. Although we adjusted for known confounders including BMI, smoking, alcohol consumption, and diabetes mellitus through multivariable regression models, the possibility of residual confounding cannot be entirely ruled out. For instance, alcohol consumption is not only a risk factor but may also indirectly alter lipid metabolism and inflammatory status through its effects on liver function. Future studies should employ propensity score matching or rigorous subgroup analyses to further validate the independent role of neutrophil count. Furthermore, as a non-specific inflammatory marker, the neutrophil count is susceptible to factors such as infection or stress, and its specificity warrants further consideration. Our study also did not examine the relationship between systemic inflammation and progression to a dilated phenotype or arrhythmic complications, which warrants investigation in future prospective studies.

Future multicenter, large-scale prospective studies are needed to validate the external applicability of this model. The main limitation is the single-center retrospective design and small sample size, particularly the few obstruction cases, risking overfitting and limiting generalizability. We plan a multicenter prospective study to externally validate the nomogram, expand the sample size, and assess its real-world clinical utility. Rigorous validation is needed before this nomogram can be considered a reliable clinical tool. Subsequent research could further explore the specific molecular mechanisms by which alcohol intake and inflammatory responses contribute to the progression of HCM obstruction. Additionally, incorporating cardiac magnetic resonance (CMR) features or genetic data to construct a multimodal prediction system could be attempted. The ultimate goal is to identify high-risk populations using this model and, through targeted clinical interventional trials, evaluate whether lifestyle modifications or anti-inflammatory therapies can effectively delay the progression from non-obstructive to obstructive HCM.

## 5. Conclusions

This study identified five independent predictors of LVOTO in HCM patients—a history of alcohol consumption, peak LVOTPG, maximum wall thickness, peak mitral E-wave velocity, and neutrophil count—and constructed an individualized nomogram prediction model. Despite its promising predictive performance (AUC 0.810–0.850), the single-center retrospective design and limited sample size necessitate cautious interpretation. Prospective external validation is required before this model can be applied clinically to assist in risk stratification and guide individualized management of HCM patients.

## Figures and Tables

**Table 1:** Comparison of baseline characteristics between the two groups.

Variable	Non-Obstruction Group (*n* = 177)	Obstruction Group (*n* = 53)	*p*
Sex (*n*, %)			0.367
Male	122 (69.0)	39 (73.6)	-
Female	55 (31.0)	14 (26.4)	-
Age (*n*, %)	62.5 (55.0; 68.0)	58.0 (43.8; 66.8)	0.069
BMI (kg/m^2^)	24.0 (22.0; 26.0)	25.0 (23.2; 29.0)	0.012
Smoking history (*n*, %)	41 (23.2)	16 (30.2)	0.0347
Alcohol consumption (*n*, %)	2 (1.1)	13 (24.5)	0.017
Comorbidities and Medication History			-
Hypertension (*n*, %)	74 (41.8)	18 (34.0)	0.763
Diabetes mellitus (*n*, %)	19 (10.7)	5 (9.4)	0.982
Coronary artery disease (*n*, %)	34 (19.2)	12 (22.6)	0.22
Lipid-lowering agents (*n*, %)	25 (14.1)	2 (3.8)	0.166
ARBs (*n*, %)	24 (13.6)	8 (15.1)	0.468
β-blockers (*n*, %)	23 (13.0)	6 (11.3)	0.991
CCB (*n*, %)	36 (20.3)	8 (15.1)	1
Diuretics (*n*, %)	8 (4.5)	1 (1.9)	1

**Table 2:** Comparison of CBC and lipid profile parameters between obstructive and non-obstructive HCM patients.

Variable	Non-Obstruction Group (*n* = 177)	Obstruction Group (*n* = 53)	*p*
B-type natriuretic peptide (BNP)	119 (50.2; 324)	193 (77.5; 434)	0.195
White Blood Cell count (10^9^/L)	6.34 (5.12; 7.44)	6.46 (5.86; 7.56)	0.285
Platelet count (10^9^/L)	188 (169; 225)	230 (186; 246)	0.031
Neutrophils (10^9^/L)	3.87 (3.01; 4.96)	4.19 (3.48; 4.90)	0.222
Lymphocytes (10^9^/L)	1.67 (1.31; 2.04)	1.82 (1.27; 2.13)	0.387
Monocytes (10^9^/L)	0.40 (0.32; 0.49)	0.42 (0.34; 0.52)	0.324
Triglyceride (TG) (mmol/L)	1.38 (0.96; 2.20)	1.67 (1.02; 3.15)	0.119
Total cholesterol (TC) (mmol/L)	4.22 (3.28; 5.03)	4.44 (3.35; 4.94)	0.533
Low-density lipoprotein (LDL) (mmol/L)	2.47 (1.90; 3.12)	2.57 (1.78; 3.34)	0.473
High-density lipoprotein (HDL)	1.01 (0.89; 1.17)	0.92 (0.80; 1.05)	0.01

**Table 3:** Comparison of inflammatory biomarkers between the two groups.

Variable	Non-Obstruction Group (*n* = 177)	Obstruction Group (*n* = 53)	*p*
NHR	3.87 (2.92; 5.11)	4.63 (3.45; 5.92)	0.031
MLR	0.40 (0.31; 0.51)	0.47 (0.36; 0.58)	0.05
LMR	1.65 (1.28; 2.21)	2.04 (1.33; 2.44)	0.096
PHR	188 (156; 240)	240 (185; 297)	0.07
SII	437 (314; 632)	596 (324; 772)	0.119
SIRI	0.95 (0.66; 1.43)	1.00 (0.76; 1.61)	0.372
AISI	175 (116; 290)	237 (150; 364)	0.099

**Table 4:** Multivariable Logistic Regression Analysis of Risk Factors for Hypertrophic Cardiomyopathy (*p* < 0.1).

Multivariate Regression	Characteristics	B.x	SE.x	OR.x	CI.x	Z.x	P.x
1	History of alcohol consumption	1.302	0.462	3.68	1.49–9.09	2.820	0.005
2	Peak LVOTPG	1.375	0.524	3.96	1.42–11.05	2.624	0.009
3	Maximum ventricular wall thickness	0.021	0.009	1.02	1–1.04	2.241	0.025
4	Peak mitral valve E-wave velocity	0.023	0.010	1.02	1–1.04	2.283	0.022
5	Neutrophil count	0.884	0.391	2.42	1.13–5.21	2.260	0.024
6	PHR	0.004	0.002	1.00	1–1.01	1.878	0.060
7	Peak pulmonary valve pressure gradient	0.008	0.005	1.01	1–1.02	1.705	0.088

## Data Availability

The original contributions presented in this study are included in the article/Supplementary Materials. Further inquiries can be directed to the corresponding author.

## Supplementary Materials

The following supporting information can be downloaded at: https://www.mdpi.com/article/10.3390/jcdd13090419/s1, Figure S1: (A,B) represent the ROC curves for the training and validation cohorts, respectively, while (C,D) show the DCA curves for the training and validation cohorts, respectively; Table S1: Comparison of echocardiographic structural and functional parameters between obstructive and non-obstructive HCM patients; Table S2: Logistic regression analysis of risk factors for hypertrophic cardiomyopathy.
