# Supplementary material for: A Nomogram Integrating Inflammatory Biomarkers and Echocardiographic Parameters for Predicting Left Ventricular Outflow Tract Obstruction Risk in Hypertrophic Cardiomyopathy
Source: J Cardiovasc Dev Dis. 2026 Aug 27;13(9):419. doi: 10.3390/jcdd13090419 (PMC13607843; doi:10.3390/jcdd13090419)
Supplement: Supplementary file 1 [file jcdd-13-00419-s001.zip › jcdd-4465353-supplementary.pdf]

Table S1. Comparison of echocardiographic structural and functional parameters between obstructive and non-obstructive HCM patients

Variable	Non-obstruction group（n=177）	Obstruction group (n=53)	*p*
Aortic root diameter (cm)	3.40 (3.10; 3.60)	3.30 (3.10; 3.60)	0.544
Left atrial diameter (cm)	3.90 (3.70; 4.30)	3.95 (3.70; 4.30)	0.333
LV end-diastolic diameter (cm)	4.50 (4.20; 4.90)	4.85 (4.60; 5.20)	0.152
Interventricular septal thickness (cm)	1.70 (1.50; 2.00)	1.30 (1.00; 2.18)	0.086
LV posterior wall thickness (cm)	1.10 (1.00; 1.30)	1.30 (1.20; 1.60)	<0.001
Pulmonary artery diameter (cm)	2.30 (2.10; 2.50)	2.30 (2.12; 2.50)	0.452
Right atrial diameter (cm)	3.60 (3.23; 4.00)	3.60 (3.20; 3.88)	0.435
Right ventricular diameter (cm)	3.20 (2.90; 3.60)	3.10 (2.80; 3.50)	0.104
Aortic valve peak velocity (cm/s)	134 (118; 160)	179 (133; 203)	<0.001
Aortic valve peak pressure gradient (mmHg)	7.00 (5.00; 10.0)	11.6 (7.00; 17.5)	<0.001
Pulmonary valve peak velocity (cm/s)	98.0 (85.0; 116)	104 (94.0; 130)	0.035
Pulmonary valve peak pressure gradient (mmHg)	4.00 (3.00; 5.00)	5.00 (4.00; 7.75)	<0.001
Mitral valve E-wave velocity (cm/s)	59.0 (49.0; 71.8)	70.0 (56.5; 80.5)	0.022
Mitral valve E-wave pressure gradient (mmHg)	2.00 (1.00; 2.00)	2.00 (1.00; 3.00)	0.027
Mitral valve A-wave velocity (cm/s)	80.0 (62.0; 94.0)	100 (69.2; 118)	0.002
Mitral valve A-wave pressure gradient (mmHg)	3.00 (2.00; 4.00)	5.00 (2.25; 6.00)	<0.001
Maximum ventricular wall thickness (mm)	1.80 (1.60; 2.00)	2.00 (1.70; 2.28)	0.005
LVOTPG (mmHg)	6.00(4.00; 12.8)	52.0(40.2; 91.2)	<0.001

Table S2. Logistic regression analysis of risk factors for hypertrophic cardiomyopathy

**Univariate logistic regression**	**Characteristics**	B.x	SE.x	OR.x	CI.x	Z.x	P.x
1	AISI	0.002	0.001	1.00	1-1	2.298	0.022
2	History of alcohol consumption	1.027	0.381	2.79	1.32-5.9	2.693	0.007
3	BNP	0.041	0.016	1.04	1.01-1.08	2.615	0.009

4	Angiotensin II receptor blockers	0.696	0.419	2.01	0.88-4.56	1.664	0.096
5	Calcium channel blockers	0.751	0.384	2.12	1-4.5	1.958	0.050
6	Peak LVOTPG	1.066	0.434	2.90	1.24-6.8	2.457	0.014
7	Peak LVOT velocity	0.044	0.035	1.05	0.98-1.12	1.249	0.212
8	Maximum ventricular wall thickness	0.017	0.007	1.02	1-1.03	2.334	0.020
9	Peak mitral valve E-wave pressure gradient	0.006	0.007	1.01	0.99-1.02	0.840	0.401
10	Peak mitral valve E-wave velocity	0.022	0.012	1.02	1-1.05	1.759	0.079
11	Neutrophil count	0.806	0.317	2.24	1.2-4.17	2.543	0.011
12	NHR	0.277	0.113	1.32	1.06-1.65	2.445	0.014
13	PHR	0.005	0.002	1.00	1-1.01	2.316	0.021
14	Peak pulmonary valve pressure gradient	0.013	0.004	1.01	1.01-1.02	3.250	0.001
15	Peak pulmonary valve velocity	-0.592	0.438	0.55	0.23-1.31	-1.352	0.176
16	Right ventricular diameter	1.128	0.492	3.09	1.18-8.11	2.292	0.022

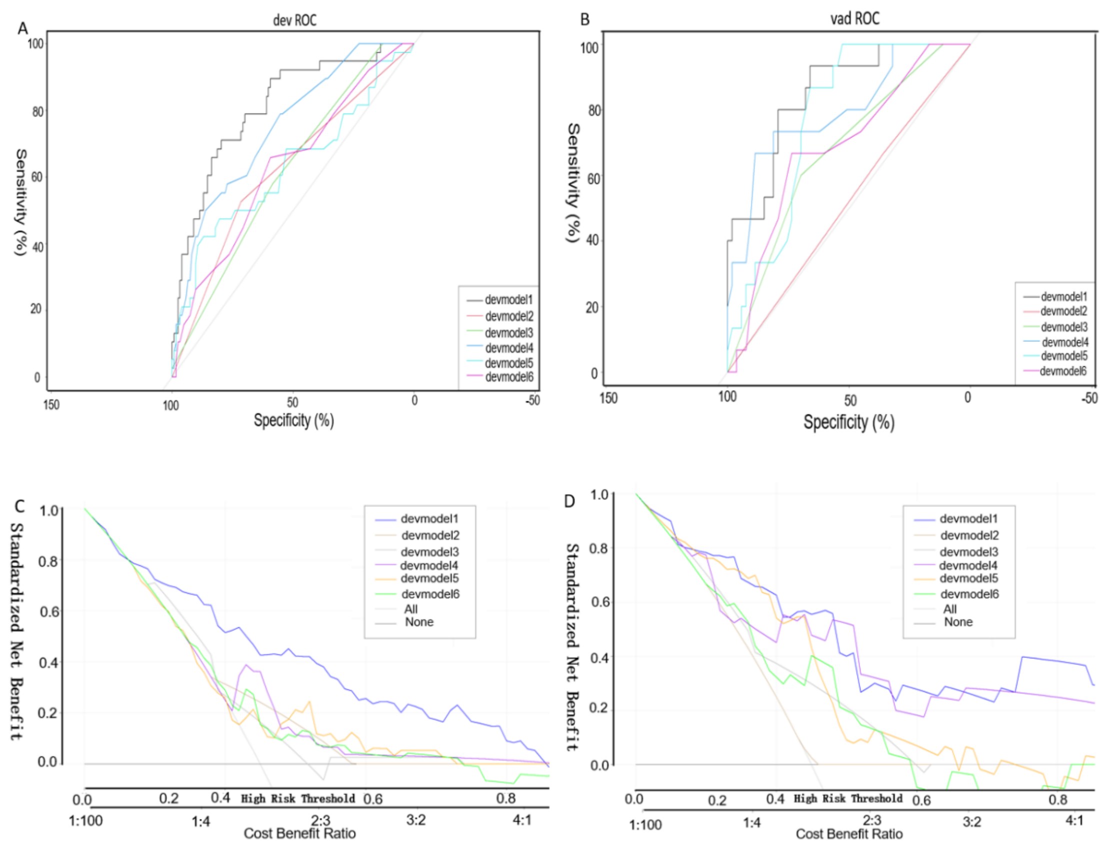

Figure S1. (**A**,**B**) represent the ROC curves for the training and validation cohorts, respectively, while (**C**,**D**) show the DCA curves for the training and validation cohorts, respectively.
